# The Roles of Nanomaterials in Conventional and Emerging Technologies for Heavy Metal Removal: A State-of-the-Art Review

**DOI:** 10.3390/nano9040625

**Published:** 2019-04-17

**Authors:** Mahesan Naidu Subramaniam, Pei Sean Goh, Woei Jye Lau, Ahmad Fauzi Ismail

**Affiliations:** Advanced Membrane Technology Research Centre, School of Chemical and Energy Engineering, Faculty of Engineering, Universiti Teknologi Malaysia, Johor 81310, Malaysia; mahesannaidu@yahoo.com (M.N.S.); wjlau@petroleum.utm.my (W.J.L.)

**Keywords:** heavy metal removal, nanomaterials, adsorption, photocatalysis, membrane

## Abstract

Heavy metal (HM) pollution in waterways is a serious threat towards global water security, as high dosages of HM poisoning can significantly harm all living organisms. Researchers have developed promising methods to isolate, separate, or reduce these HMs from water bodies to overcome this. This includes techniques, such as adsorption, photocatalysis, and membrane removal. Nanomaterials play an integral role in all of these remediation techniques. Nanomaterials of different shapes have been atomically designed via various synthesis techniques, such as hydrothermal, wet chemical synthesis, and so on to develop unique nanomaterials with exceptional properties, including high surface area and porosity, modified surface charge, increment in active sites, enhanced photocatalytic efficiency, and improved HM removal selectivity. In this work, a comprehensive review on the role that nanomaterials play in removing HM from waterways. The unique characteristics of the nanomaterials, synthesis technique, and removal principles are presented. A detailed visualisation of HM removal performances and the mechanisms behind this improvement is also detailed. Finally, the future directions for the development of nanomaterials are highlighted.

## 1. Introduction

In a bid to bolster its economic growth, heavy industrialization across developing countries is rampant. One hindsight with this phenomenon is the creation of many types of poorly managed waste that eventually seep deep into the environment via air, soil, and water. The damage of natural resources takes place as the direct consequence of the release of hazardous substances [1]. In particular, water is the prominent recipient of many types of pollutant. The continuous pollution of water, in tandem with the increased demand for fresh water due to industrialization and population growth, has strained water resource to a breaking point. Various types of pollutants, such as natural organic matters (NOM), oil, pathogens, and heavy metals (HM) have badly afflicted water. HM pollutions has been a prominent issue, as the HM infusing into the water sources can be produced from various human activities, including mining, agriculture, and electronic industries [2]. It is imperative that HM contents in waterways are controlled efficiency in view of their poisonous and toxic nature towards all organisms. In a broad context, HMs are a group of trace metals with an atomic density of less than 5 ± 1 g/cm^3^ [3]. Cadmium, manganese, iron, arsenic, and mercury are some of the prominent HMs that are commonly found in wastewater [4]. These metals usually exist in the form of ions in waterways and soil. They can pose a health hazard to both humans and the ecosystem via avenues, such as direct ingestion or in contact with contaminated water or soil, drinking water that has been contaminated with HM, ingestion of foodstuff laden with HM (plants or aquatic life), as well as the accumulation of HM via the food chain. Critically, organisms are unable to metabolize and excrete HMs out of the body. The presence of HM ions in water samples imparts great ecological impact due to its toxicity and bioaccumulation, as aquatic life is known to accumulate significant concentration of metals in water where the presence of such metal in water samples are below the detection levels [5].

The sources of HM ions in the environment can be focused on two origins, i.e., natural sources and human activities. The former source includes phenomena, such as landslides, weathering, and volcanic eruption. These activities have significantly contributed to HM pollution, as these events release trapped HM ions into the environment. Anthropogenic activities are also a major source of HM pollution. Activities, such as mining, smelting operations, industrial production and manufacturing industry, and agricultural use of HM in the form of pesticides are major causes of HM pollution on soil and in water. Controlling the leaching of HM into the environment is important. Various legislations and laws have been enacted to control the number of pollutants that are released by industry players into the environment. Table 1 shows the maximum level of HM content in water samples across different local and global agencies.

On the other hand, the remediation of these HMs is also essential in reducing the impact of HM on human health. As of now, numerous strategies have been developed to separate these HMs from water sources. Remediation techniques include the employment of absorbents, coagulation of HM ions, chemical precipitation, membrane filtration, electrodialysis, and photocatalysis. Each of these techniques has its unique advantages as well as disadvantages that are particularly associated to their efficiency for large scale HM remediation. Unique nanomaterials that were developed by researchers are at the center of these treatment methods. The discovery and the subsequent extensive research on nanomaterials developed in different geometries have brought about different physicochemical properties that cannot be expressed by bulk materials. Nanomaterials, such as carbon nanotubes (CNT) [7], graphene [8], titania nanotubes (TNT) [9], hybrid metal-non-metal nanomaterials, such as graphite silica [10] and graphene oxide-magnetite nanocomposite [11], and other metal oxide-based nanomaterials have been extensively used to remove HM ions from polluted waters, with a varying degree of success. These nanomaterials can be advantageously used to remove HM ions via adsorption, owing to their large adsorption capacities. Especially, nanomaterials of different geometries have exhibited high HM removal efficiency due to unique properties, such as large surface area, specific surface charge values, surface functionality, and HM ions binding capabilities. These nanomaterials can also be incorporated with other treatment techniques, such as adsorptive membranes and composite membranes to work synergistically and to further improve HM ion removal efficiency.

Over the years, a number of comprehensive reviews have been made in regard to nanomaterials and their applications for environmental remediation. Khin et al. compiled a comprehensive review of the application of nanomaterials as a viable solution in the removal of various types of pollutants and biological contaminants [12]. Azzouz et al. published a more recent review on the utilization of nanomaterials as sorbents in a solid-phase pollutant extraction system for environmental samples [13]. The author discussed the use of various types of nanomaterials as potential sorbents for analytical applications. Jeevanandam et al. prepared a review on the history, sources, toxicity, and regulations of nanoparticles and nanostructured materials [14]. Despite the efforts that have been made in this area, very small number of reviews has been made to discuss the significant role of nanomaterials in removing pollutants from wastewater [15]. It is worth providing insights regarding the functionality of nanomaterials of different compositions, characteristics and structures, as well as their key roles in the removal of HM ions, particularly in different remediation techniques from various sources.

In this paper, an overview of prominent sources and types of HM containments in the water sources is first provided. Next, various nanomaterial synthesis techniques were used to prepare unique nanomaterials especially for HM ions removal from water sources are discussed. In the main body of this review, the performances of nanomaterials in various HM removal strategies are evaluated. Finally, the current hurdles and future directions of the HM removal strategies that are based on nanomaterials are highlighted.

## 2. Heavy Metal Ions in the Environment

### 2.1. Sources of Heavy Metal

HMs have great ecological consideration, due to their toxicity and accumulation. Fish might accumulate significant concentrations of metals in water in which those metals are below the limit of detection in a routine water sampling and analysis [16]. The sources of HM in the ecosystem are focused on human activities and natural phenomena. Some of the sources include the usage of HM-laden pesticides and naturally occurring HM from the Earth’s core via activities, such as soil erosion and volcanic eruption.

Pesticides (ethylene dibromide, and methyl bromide fungicides), insecticides (dithiocarbamates, and captan), and herbicides (paraquat, diquat, and 2, 4-dichlorophenoxyacetic acid) have been used for many decades as means to improve the survival of vegetations that are planted for commercial purpose and used to kill off various kinds of pest that harm the quality of crops. HMs are usually used as active compounds in the aversion of pest. Copper, usually in the form of copper sulfate and mercuric chloride, is used for its anti-fungicidal properties. Sodium, in the form of sodium dichromate works as a cotton defoliant [17]. Zinc phosphide is used as a rodenticide, whist cadmium chloride is used as a fungicide [18]. These pesticides laden with HM are usually prepared in ionic form, which later dissociates after being dispersed into the soil. Their nature allows for them to reside in water and travel far away from the point of origin. Pesticides can be taken up by plants, dissociate into soil, or carried away by residual water into other water bodies in contact, such as rivers and lakes [19]. Absorption of pesticides by plants removes them from the environment. However, plants cannot metabolize these compounds and stay in the plants, which can be transferred to other organisms that consume these plants, such as animals and humans itself [20]. Evidence of HM ending up in plant specimens is aplenty [21,22,23]. Via phytoextraction, plants can absorb HMs, which are essential in plant growth [24]. However, they also absorb HMs, such as cadmium, chromium, and lead, which do not serve them in any biological function. Consequently, the bioaccumulated HM will be passed along the food chain [25].

### 2.2. Effect of Heavy Metal

#### 2.2.1. Effect of Heavy Metal Ions Towards the Environment

**Figure 1:**
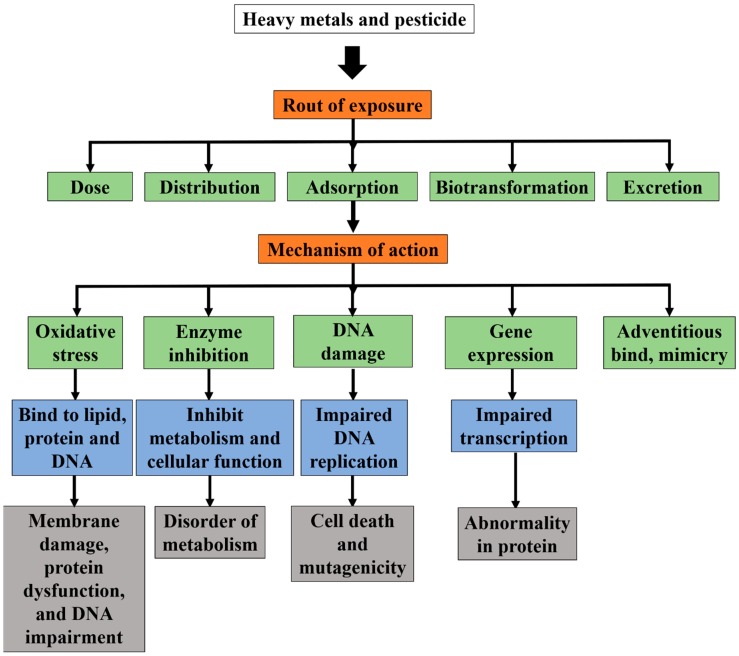
Common route of absorption, distribution, and excretion related to the exposure of HMs and inorganic pesticides. Adapted from [29], with permission from Frontiers, 2017.

The largest contributor of HM in the air is the usage of hydrocarbons, such as gasoline, diesel, and petrol. HM, such as arsenic, lead, and cadmium, are emitted when these hydrocarbons are combusted [26]. Volcanic eruptions produce hazardous impacts to the environment, as the deterioration of social and chemical conditions and the gases (carbon dioxide, sulfur dioxide, carbon monoxide, and hydrogen sulfide) that are released during eruptions, various organic compounds and HMs, such as mercury, lead, and gold, are also released [4]. HMs enter plant and animal tissues via air inhalation, diet, and manual handling. Motor vehicle emissions are a major source of airborne contaminants, including arsenic, cadmium, cobalt, and nickel. HMs leaching from industrial and consumer waste can pollute water sources (groundwater, lakes, streams, and rivers); acid rain can exacerbate this process by releasing HMs that are trapped in soils. However, the risk of these metals entering the food chain is highly dependent on the mobility of the metal cations and its bioavailability in soil. The metal cations are bound to negatively charged particles in soil, such as clay and organic matter. When these metal cations detach from the negatively charged particles, they become available to be absorbed by plants and other organisms that live in the soil [25]. Plants are exposed to HMs through the uptake of water, and are stored until animals, which then transfer the HM into the animal’s body, consume these plants. The ingestion of plant and animal-based foods that are laden with HM is one of the worrying sources of HMs in humans. The presence of such inorganic pesticide can also degrade the soil due to the accumulation of compounds at undesirable levels [27]. Absorption through skin contact with soil is another potential source of HM contamination. Studies have also shown that HMs can be accumulated in the plant tissues of *Sebera acuminate* and *Thlaspi caerulescens,* as they cannot be metabolized [21]. Arsenic poisoning is one of the most prevalent HM cases across the globe, which usually occurs by drinking water that is contaminated with arsenical pesticides, natural mineral deposits, or inappropriate disposal of arsenical chemicals. A work done by Sim et al. revealed that rivers in Sarawak have experienced extensive land use and logging, and hence suffer from contaminations of HMs, such as arsenic, chromium, and copper [28]. Another similar research also revealed that River Pra and its tributaries displayed an enrichment in HM ions [2]. Figure 1 shows the route of absorption, distribution, and excretion that are related to the exposure of HMs and inorganic pesticides [29].

#### 2.2.2. Effect of Heavy Metal Ions Towards Humans

Human can be afflicted with HM poisoning due to the consumption of food or water that is laden with HM. They stay in the human body system and result in constant accumulation of different types of HM, ince humans are not able to metabolize it. Exposure to As (V) leads to an accumulation of As (V) in tissues, such as skin, hair, and nails, resulting in various clinical symptoms, such as hyperpigmentation and keratosis. There is also an increased risk of skin, internal organ, and lung cancers. Lead is known to disrupt the balance between the production of free radicals and the generation of antioxidants to detoxify the reactive intermediates or to repair the resulting damage [30]. Reactive oxidation species (ROS) may cause structural damage to cells, proteins, nucleic acid, membranes, and lipids, resulting in a stressed situation at cellular at very high concentrations [31]. Lead is also known to disrupt biological processes, such as cell adhesion, intra- and inter-cellular signaling, protein folding, maturation, apoptosis, ionic transportation, enzyme regulation, and release of neurotransmitters [32]. Aluminium is a common HM that is used in the production of carbonated drink cans and cooking utensils [33]. The WHO postulated that aluminium exposure is probably a risk factor in the onset of Alzheimer disease in humans, whist reports have also suggested that humans can also be afflicted with contact dermatitis and irritant dermatitis [30,34]. Mercury, as a type of HM that is commonly found in many types of seafood and being previously used in dental amalgam, can also potentially harm humans due to their acute toxicology. Mercury tends to be tightly bound in the brain, spinal cord, ganglia, autonomic ganglia, and peripheral motor neurons upon entering into the human body [35,36].

### 2.3. The Chemistry of Heavy Metal Ions

HM ions tend to present in salt or oxide forms. The HM ions ionic values are generally between +2 to +6, which indicates that metals placed in group 2 to group 6 of the periodic table of elements are categorised as HMs. Differing ionic states of HMs can significantly affect the suitability of different HM removal techniques and their conditions or parameters. For instance, the ionic state of HM can be altered in different pH conditions, which in turn affects the electrostatic interaction between the nanoadsorbent and the HM ions [37]. For the removal of HM via adsorption, the pH condition between 4 and 7 is favourable, as it greatly improves surface coordination, electrostatic attraction, and co-precipitation, which will result a higher removal rate. Increment from pH 4 to 7 reduces protonation phenomena, as it increases the H^+^ availability. As the pH increases, the overall charge positivity increases and it also increases the interaction between the HM and adsorbent. This also increases the formation of chelate complexes between metal cations and the lone pair of electrons on the sulfur and nitrogen atom [38]. HM ions, such as Pb (II) and Hg (II) are efficiently adsorbed by neutrally charged adsorbents in the pH range between 4 and 7. On the other hand, when the absorbent surface is charged, the pH can also be manipulated to attain a parameter where the greatest electrostatic interaction between adsorbent and HM occur. Besides, the surface functional group also plays a role in the adsorption capacity. Al-Senani et al. exhibited that the removal of Co (II) and Cd (II) was effective above pH 9, exhibiting a removal rate of more than 98% [39]. This is due to the functional group exhibited by the adsorbent, which includes carboxyl, hydroxyl, and amine, all being involved in the binding mechanism between adsorbent and HM.

## 3. Nanomaterials-Assisted Approach for Heavy Metal Removal

The removal of HM can be carried out by several traditional techniques. Some of the methods include electrochemical treatment, photocatalysis, coagulation, adsorption, chemical precipitation, and membrane technologies, such as reverse osmosis and nanofiltration. However, in this review paper, three main HM removal technologies that have portrayed the greatest potential for large scale application have been focused upon. The HM removal techniques in focus are adsorption, photocatalytic reduction and membrane filtration/adsorption.

### 3.1. Adsorption of Heavy Metals

**Figure 2:**
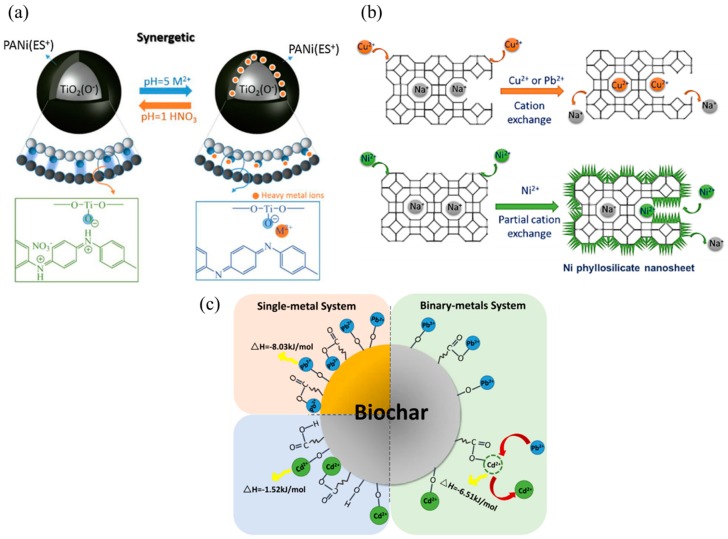
Schematic illustration of the adsorption of HM via the surface of (**a**) hybrid polyaniline/TiO_2_ nanocomposite adsorbents. Adapted from [45], with permission from Elsevier, 2018. (**b**) cation exchange by hierarchically porous zeolite for improved adsorption of cationic HMs. Adapted from [46], with permission from Elsevier, 2019. and (**c**) selective HM ion adsorption by biochar in a single and binary metal system. Adapted from [47], with permission from Elsevier, 2019.

Adsorption is a process where a surface holds a molecule onto it. Adsorption happens via two phenomena, which are physisorption or chemisorption [40]. IUPAC defines adsorption as the increase in concentration of a dissolved substance at the interface of a condensed and a liquid phase due to the operation of surface forces [41]. Physisorption occurs when forces, such as intermolecular forces, are used to attach the absorbate onto the absorbent meanwhile chemisorption involves valence forces of the same kind as those operating in the formation of chemical compounds [42]. For any adsorption process, kinetic and isotherm studies are performed to evaluate the adsorption phenomena, rate, and efficiency [43]. Some of the common parameters that affect adsorption include the pH of the aqueous solution, the interaction between the adsorbent and adsorbate, surface charge, the surface area of adsorbate, and the size of adsorbent and adsorbate. Adsorption is a viable way of removing HM from water bodies. Many researchers have conducted a study investigating the ideal pH value, adsorbate surface area, and porosity and surface charge for effective adsorption of various types of HM ions [44]. Figure 2 illustrates the possible interaction of HM ions with the surface if adsorbents, such as polyaniline/TiO_2_ composites [45], cation exchanged porous zeolite [46], and binary metal adsorption by biochar derived from activated sludge [47].

### 3.2. Photocatalytic Reduction of Heavy Metal

**Figure 3:**
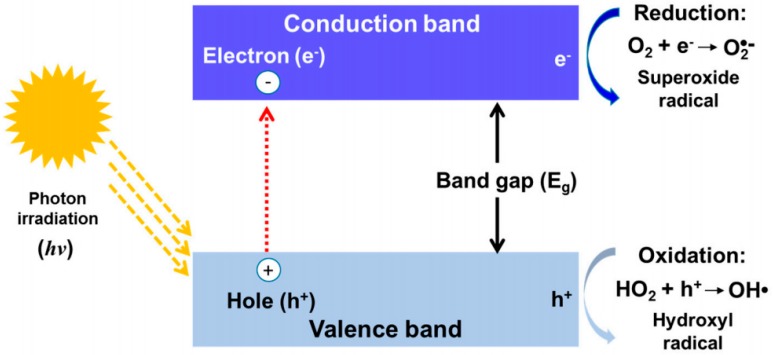
Excitation of an electron in a structure of photocatalyst and subsequent creation of ROS. Adapted from [48], with permission from Elsevier, 2018.

Among many types of wastewater remediation techniques that have been discovered by researchers, photocatalysis remains one of the best methods, simply because it is able to destroy or reduce the pollutant, rather than just mitigate, trap, or isolate them. The application of various types of semiconductor materials, including titanium dioxide (TiO_2_) and zinc oxide (ZnO) as a light responsive material to treat wastewater that is laden with organic have garnered much of the attention of emerging researchers. These semiconductors can produce strong oxidative free radicals that are capable of destroying a large range of organic pollutant and reduce HM ions when it is irradiated with light sources. These semiconductors harvests the photons and excite electrons into a higher energy state when light is irradiated, producing electron pair holes that are transferred on the surface of the semiconductor, which in turn, produces these ROS such as OH● and O_2_^●−^. Figure 3 shows a brief illustration on the excitation of an electron in a structure of photocatalyst and the subsequent creation of ROS species.

Many types of research have been focused on the fine-tuning and the modification of these semiconductors for efficient degradation of organic pollutants since semiconductors are able to efficiently degrade NOM. The possibility of using photocatalyst in the removal of HM is less explored, as it is impossible to degrade metallic ions. However, photocatalyst has shown promise as a means to reduce the harmful effects of HM by reducing the metal ions into less harmful by-products. The reduction of HM ions is a viable means for the treatment of HM pollution. Cr (VI) is significantly harmful to organisms, even in small trace amount, as compared to Cr (II). Hence, the common practice for remediation of Cr (VI) is reducing it to Cr (II). This is where photocatalysts can play a prominent role in HM waste remediation. Based on the current research trend, the employment of photocatalyst has been commonly used as means to reduce Cr (VI), which can be abundantly found in contaminated water, sourced from industries, such as electroplating, pigmentations, and so on [48]. The photoreduction of Cr (VI) is elucidated in Equation (1) and Equation (2) [49];

Cr_2_O_7_^2−^ + 14H^+^ + 6e^−^ → 2Cr^3+^ + 7H_2_O (1)

2H_2_O + 2h^+^ → H_2_O_2_ + 2H^+^ (2)

Cr (VI) has been extensively researched, because the hexavalent equivalent is much more poisonous than its divalent variant, Cr (III) counterpart. When the photocatalyst is irradiated with photons, they absorb it and excite an electron towards the particle surface. The Cr (VI) ions consume these electrons, allowing single photoreduction. This, in turn, makes the catalyst reactive due to the continuous presence of an electron-hole pair. To exploit this mechanism, research regarding photoreduction of Cr (VI) metal ions are paired with an organic pollutant, as the organic pollutant becomes an electron source for the electron deprived photocatalyst, which initiates a chain reaction on continuous photoreduction of Cr (VI) and the degradation of an organic pollutant. This mechanism provides further proof that photocatalysis can work for the photodegradation and photoreduction of wastewater mixed with HM ions and organic pollutants, hence enhancing its versatility [49].

### 3.3. Membrane Filtration/Adsorption

**Figure 4:**
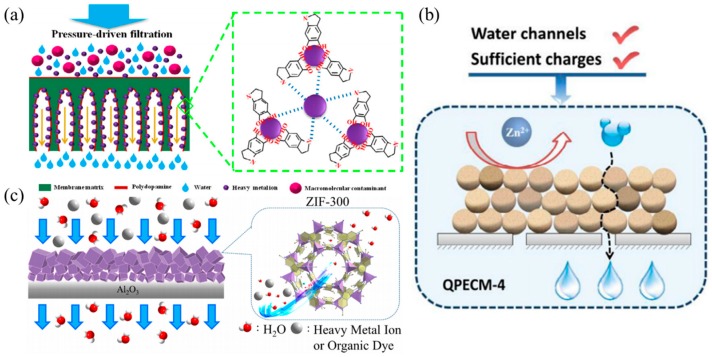
HM removal via (**a**) adsorptive membrane technique. Adapted from [56], with permission from Elsevier, 2017. (**b**) surface-charged modified membrane repellent. Adapted from [57], with permission from Elsevier, 2019. and (**c**) size exclusion of HM ions. Adapted from [58], with permission from Elsevier, 2019.

Membrane separation is another emerging technology that has shown great promise in HM separation from polluted waters. The focus for development of a membrane-based solution for HM remediation intensified when researchers discovered problems in other common remediation, which include the poor reusability of adsorbents, high cost of material development, and difficulty in separating nanomaterials from the water after remediation [50]. The employment of membrane-based remediation method can address these problems, as membrane that is incorporated with novel nanomaterials is a one-step method that can be reused while maintaining excellent rejection of HM in aqueous solution without the need of chemicals or pre-treatments. In the search for the development of membranes that are low in cost, high reusability, greater selectivity, better water transport, and high HM ions rejection, researchers are exploring the possibility of incorporation of various types of nanomaterials in a bid to impart the unique characteristics of nanomaterials into membranes. The addition of high surface area nanomaterials has developed highly adsorptive membranes [51], while the incorporation of highly hydrophilic nanomaterials on the membrane surface has significantly improved the water permeability of membranes [52]. Photocatalytic hybrid nanomaterials, such as Graphitic carbon nitride (g-C_3_N_4_) quantum dots (QD) [53] and TNT array [54], are also developed to allow for simultaneous photocatalysis and membrane filtration in efficient wastewater treatment [55]. Figure 4 shows the utilization of several membrane-based technologies in the remediation of water bodies laden with HM ions [56,57,58].

Even though there are many ways that membranes are employed to remove HM, including surface charged membranes for HM ion repellent, membrane distillation [59], adsorptive membrane [60], size exclusion removal [57], and more, there are two popular ways where HM can be removed from water bodies using membrane-based remediation, which is size exclusion removal, or using adsorptive membranes [61]. Commonly, membrane works by sieving the molecules according to size. Only particles that are larger than the pore size are retained. In this case, the removal of HM requires the employment of nanofiltration (NF) membranes, as the pore size of common ultrafiltration (UF) membranes are large and would allow HM ions to pass through. However, NF membranes face one prominent problem. They will restrict the movement of water through the membrane since the pore size is very small, which drastically reduces membrane flux [62]. To overcome this, researchers have explored the idea of adding novel nanomaterials into these membranes to improve the permeation, whilst maintaining or enhancing the HM rejection rate. Nanomaterials, such as halloysite nanotubes [63], CNT [64], metal oxide nanoparticles [65], and many more are blended into a polymeric matrix or are deposited on the membrane surface to improve selectivity and permeation, whilst maintaining or enhancing rejection capabilities. The improved performance of membranes is owed to the modification of characteristics due to the presence of nanomaterials. Materials, such as CNT’s, create a pathway for water transport through the membrane matrix, increasing water permeation [66]. Other nanomaterials, such as amine grafted SiO_2_, are able to impart superhydrophilicity into a polymeric membrane, which is generally hydrophobic in nature [67]. Adsorptive filtration is another way where membranes can be employed for HM removal. Albeit, adsorption itself is a promising way to treat HM waters, it is bugged by a few problems. Sorbents always tend to float on the water surface, and this would render proper mixing with water to achieve maximum contact between adsorbent and HM ions untenable [68]. If the adsorbents are properly mixed, then their form as a particle would require a form of method to separate the used adsorbent from the water source [69]. This would incur additional time and resource. Hence, adsorptive membranes have been developed to overcome this, where potent adsorbents are immobilized into the membrane matrix [70]. Adsorptive membranes exhibit the ability to trap the HM ions and at the same time, allow the filterability of water, and produce clean permeates with the metal trapped in the membrane matrix. Commonly, UF membranes are used as an adsorptive membrane, as it has a larger pore size as compared to the NF membranes. To overcome the trade-off for the low rejection of HM ions, nano-adsorbent is incorporated into UF membranes to maintain both high membrane flux due to the larger pore size and excellent HM removal.

## 4. Nanomaterials for Heavy Metal Removal

Adsorption has shown the greatest potential in terms of cost and effectiveness, even though all of the techniques are able to perform admirably in removing HM from water sources, while membrane filtration techniques have also shown good performance with long term stability and usage [62]. In addition to this, photocatalysis, have shown significant promise, owing to its non-selective degradation, excellent ability to mineralise pollutants, and good reusability [71]. Nanomaterials have played very significant roles in advancing the HM removal technologies in all of the methods that were mentioned above. Nanomaterials are described as a material with a length of 1 to 100 nm in at least one dimension [14]. Their small size allows for them to exhibit unique properties that are not shown in bulk, with some examples of properties including increased surface pore and surface area [72], improved electrical properties, increased material strength and conductivity, and self-cleaning properties [73]. Nanomaterials can be classified into metallic, non-metallic, and their composites. Metal oxides and semiconductors are common sources of metallic nanomaterials, whereas non-metallic nanomaterials include carbonous nanomaterials, such as (CNT) [74] and graphene. Nanocomposites can be multiphase nanomaterials, where one part is defined as a nanomaterial in terms of size, whilst the other may also be a nanomaterial or they can be materials that are larger with bulk-type property. In addition to this, nanomaterials are also known according to the shape that they exhibit. Some of the nanomaterial shapes include nanoparticles [75] (spherical/globular), nanosheets [76], nanowires, nanoflowers [77], nanotubes [78], and nanorods [79].

### 4.1. Motivation of Using Nanomaterial for Heavy Metal Removal

The utilization of nanoparticles for the remediation of environmental problems has shown remarkable potential in line with the rapid development of nanoscience and nanotechnology. The modification in atomic level to produce particles that are independent in nanoscale has provided a myriad of novel characteristics that cannot be found in bulk materials. Nanomaterials can be synthesized using bottom-up approach to carefully tailor the desired properties, such as surface charges and functionalities to interact with HM. Nanostructured materials have shown exceptionally high surface area and porosity, higher efficiency as an absorbent due to their superior surface to volume ratio, improved solubility, abundant reaction sides, photocatalytic properties, great surface charge, and lighter in weight or mass. These nanomaterials can also be modified via various techniques, such as surface grafting and gamma irradiation, to increase its surface reactivity [80]. CNT has also been used as a prominent adsorbent for HM removal due to their high surface area to volume ratio and their highly tunable characteristics. Various functional groups, such as hydroxyl and carbonyl groups, which can provide new adsorption sites, are easily tuned on the surface of CNT [81]. Clay can also be utilized as an efficient absorbent of HM via nanoscience. Studies have shown that common clay that is combined with activated carbon, another low cost and a common material, are able to absorb HM ions, such as Cd (II), Ba (II), and Cu (II) from pulp wastewater [82]. The synthesized absorbent was able to exhibit a surface area of close to 800 m^2^/g, which is ten-fold larger when compared to individual nanomaterial. Another research also showed that the creation of nanomaterial via a nanocasting process using mesoporous hybrid material with ZnO and TiO_2_ exhibited a surface area between 120–332 m^2^/L [83]. The absorbent also showed that it could be reused up to three times due to the micrometer-sized structure with high surface area, which has the benefit of reducing the overall cost in the adsorption process. In addition to this, semiconducting nanomaterials, which are also known as photocatalyst, can be employed in HM reduction. This is based on the fact that it has good optical properties and the energy band can be easily modified through facile modification or hybrid to render improved properties, such as lower bang gap energy, lower recombination rates, and larger active sites for photocatalysis. Nanomaterials of unique features can also be incorporated with other technologies to create a synergistic improvement in HM removal. For instance, the incorporation of novel nanomaterials into polymeric membrane matrices, such as CNT, TiO_2_, and hydrous manganese dioxide (HMO) can increase the pathways for water transport, impart photocatalytic activity, and increase membrane hydrophilicity respectively. These nanomaterials can also deposit onto the membrane surface that can greatly govern the selectivity of membranes, something that is not possible with polymers alone. The utilization of nanoscience also enabled the creation of functional nanomaterials from waste source, such as the formation of a biogenic iron (Fe) compound at a size of 500 nm, using a natural microbial consortium that was sourced from an abandoned mine containing iron oxides (Fe_2_O_3_) and siderite by bioreduction of ferric citrate [84].

### 4.2. Classification of Nanomaterials

**Figure 5:**
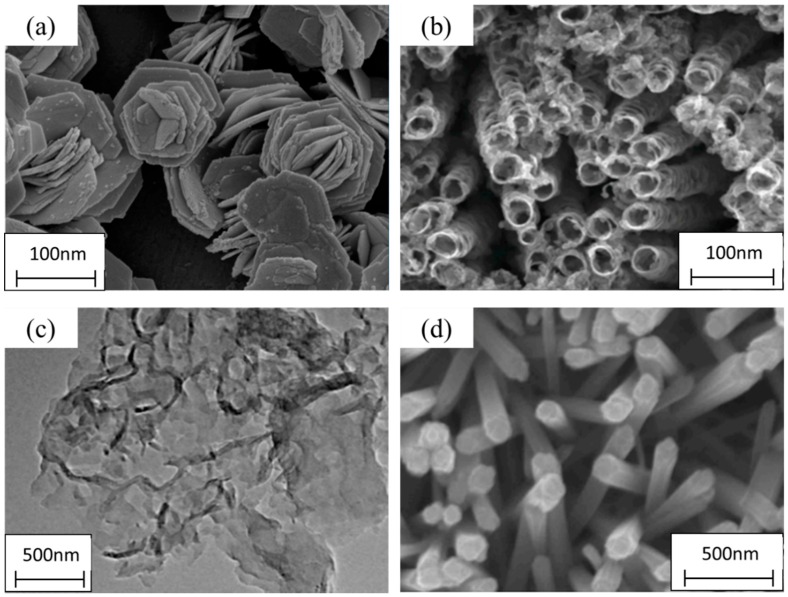
Examples of nanomaterial structures (**a**) nanoflowers. Adapted from [85]. (**b**) nanotubes. Adapted from [86]. (**c**) nanosheets. Adapted from [87], and (**d**) nanorods. Adapted from [88].

As researchers further pushed the boundary of nanoscience in the development of novel, functional materials, they discovered that the shape of nanomaterials could be manipulated. The differing shapes opened a vast array of new and unique characteristics that were not possible in its benign shape. Carbonous materials led the way, where carbon was used as the building block in building two different variants, namely CNT and graphene [76]. CNT follows the shape of tubes, while graphene took the shape of sheets, in multiple layers. Other forms also emerged, which includes the formation of nanorods and nanoflowers of different, as shown in the micrographs in Figure 5 [85,86,87,88].

The change in structure can bring about new and unique features that otherwise are not exhibited in the bulk phase. CNT’s are produced when sheets of carbon, which are called graphene, are rolled up to produce single-walled CNT’s or multi-walled CNT’s [89]. The formation of tubular structure allows graphene to be 400 times stronger than steel, allowing for conducting electricity and even working as a semiconductor, something that is not possible in bulk carbon [90]. This allows CNT’s to be cheaper and more environmentally stable materials in the development of electrical and electronic products that rely heavily on rare earth metals. TiO_2_ is a metal oxide that can be used in paints and personal cosmetic products as pigments due to its stable and environmentally safe nature. TNT have been developed and it has shown great promise in gas sensing and increased photocatalytic activity [91]. The development of tubular and porous structure, such as nanotubes and hydrated manganese oxide nanoparticles, have shown great improvement in terms of effective surface area that can reach the region of 400 m^2^/g, which is a ten-fold increase [92]. Higher surface area leads to increased reaction sites, which is valuable in the catalytic industry. Nanoflowers have been developed from materials, such as TiO_2_ and Fe_2_O_3_, exhibiting a superb volume to area ratio, better charge transfer, carrier immobility, and an enhanced number of adsorption sites [93]. All of these characteristics can significantly contribute to the field, such as drug delivery, catalytic process, chelation, and adsorption of HM ions [85]. In addition to these structures, nanorods/nanowires have also attracted the attention of researchers. Particularly, gold nanorods have received extensive attention, owing to their extremely attractive applications in biomedical technologies, plasmon-enhanced spectroscopies, pollutant remediation, and optical and optoelectronic devices. Copper nanoflowers have been shown to exhibit impressive adsorption of Pb (II) in aqueous solution, owing to its porous, high surface area structure, which significantly increases the availability of active sides and the presence of carboxylic (COOH) functional groups [94]. The flower structure is useful in drug delivery, as efficient drug deliveries require a carrier that is non-hydrolysable, controlled release pattern, and reducing drug toxicity. Nanoflowers tick all of these boxes, as displayed by the efficiency of sodium alginate/chitosan nanoflowers in drug delivery. Table 2 shows the types of nanomaterials and the important features that they exhibit as compared to the bulk material.

There are two ways where nanomaterials are structured, which can use a top-down or and bottom-up approach [107]. The top-down approach uses synthesis methods, such as lithography etching and exfoliating. This method is sparsely used due to the lack of versatility. Commonly, the bottom-up method is used, as it allows for control of the structure of nanomaterials at an atomic level, as different parameters can be manipulated to design the nanomaterial structure according to desire. Some of the common bottom-up methods in the design of nanomaterials include wet chemical precipitation, sol-gel, chemical vapor deposition, hydrothermal, sputtering, template growth, electrospinning, and atomic layer deposition [108]. In general, all of the techniques mentioned exhibit similar characteristics, which include high control towards to growth of structure, an abundant selection of precursors/starting material, control towards heat and temperature, high purity, and uniformity [109]. Section 4.3 greatly discusses the synthesis of nanomaterials.

#### 4.2.1. Metallic Nanomaterials

Metallic nanomaterials are formed from metal sources, such as titanium, iron, silver, gold, manganese, copper, and many more [110]. These nanomaterials can be produced in pure metallic form or in the metal oxide forms. Metal oxides are more favourable, as they exhibit increased stability over pure metal nanomaterials. Research that was conducted on the synthesis of nanomaterials using metals is aplenty based on current literature [111,112,113,114]. Fe_2_O_3_ is one of the commonly used metal oxide nanoparticles. Iron can work as an adsorbent for wastewater laden with HM ions. Castro et al. produced a biogenic iron compound using metal compounds that were sourced from mining wastewater via the bioreduction of ferric citrate [84]. The biogenic nature of iron compounds has high specific surface areas and high binding energies hence act as efficient adsorbents for HMs. In addition, the bacterial matrix surrounding the iron nanoprecipitates can bind harmful metals. The metal exhibited a surface area of 56.978 m^2^/g and a pore size of 8.304 nm. The large pore size allows access towards the more reactive sides to improve binding and capture the HM ions. Gold nanorods have been extensively studied due to the fact that they exhibit excellent plasmon-enhanced spectroscopies and optical and optoelectronic applications, which are hugely beneficial in the detection of HM in water samples via the colorimetric detection technique [63,77,115]. Many researchers have continuously studied upon its characteristics and its application for environmental remediation ever since the discovery of TiO_2_′s ability to split water [116]. TiO_2_ in the form of anatase crystallinity exhibits great photocatalytic activity under ultraviolet (UV) light irradiation. The good crystallinity, together with its low band gap value (3.2 eV) and stable recombination rate, makes it an excellent candidate to reduce HMs into less harmful configurations (Cr (VI) to Cr (III)) and degrade various organic pollutants [117].

#### 4.2.2. Non-Metallic Nanomaterials

The development of quantum dots is currently on the rise due to their superior features, such as edge morphology and increased in surface functional groups. Research that was done by Abdelsalam on the development of graphene quantum dots (GQDs) showed improved surface chemical functionality and well-defined edges, which is better when compared to the sheet structure that was exhibited by graphene [118]. GQD’s adsorption capability of hydrated Cd (II) and Pb (II) calculated via density functional theory (DFT) showed that it was able to absorb the hydrated HMs through different positions and interactions, including edge and surface adsorption, interaction with unsaturated carbon atoms, and adsorption on the edge of the functionalized group. The edge of surface adsorption interaction is non-existent with other nanomaterials, which shows the uniqueness of GQDs [118]. Another example of efficient non-metallic adsorbent produced from waste is the development of CNT in the adsorption of Cd (II), Cu (II), Pb (II), and Hg (II) via chemical vapor deposition (CVD). CNT developed exhibited increased surface area and porosity, with evidence of abundant functional groups, such as O-H, C-H, C=O, C-N, C=N, phenols, aromatic rings, and aromatic groups, which provides high potential for the adsorption of HMs [47]. The developed CNT was able to act as an absorbent also shows promise, as the presence of a various functional reactive group, together with the porous structure and large network of functional group, allows it to perform well as an adsorbent for HMs, such as Cu (II), Cd (II), and Pb (II) [119].

#### 4.2.3. Hybrid Nanomaterials

Hybrid nanomaterials can include only metallic materials and a mixture between metallic and non-metallic. Hybrid nanomaterials are strongly pursued due to the fact that these hybrid nanomaterials are able to exhibit improved, synergistic, or new properties that are not found in singular nanomaterials, metallic or non-metallic. Sharma et al. synthesized a metallic hybrid consisting of ZnO and TiO_2_ hybrid monolith absorbents via nanocasting, followed with calcination at 400 °C for 5 h. The hybrid nanomaterial proved to be a better adsorbent when compared to ZiO or TiO_2_ individually, as it improved the adsorption of Pb (II) and Cd (II) by more than 50% [83]. A hybrid material was developed with different ratios of clay, activated carbon and zeolite showed promising results for the adsorption of three different types of HM (Cd (II), Ba (II), and Cu (II)), which was produced via calcination [82]. Fu et al. developed a hybrid nanomaterial consisting titanate and lignin for the adsorption of Pb (II), Cu (II), and Cd (II) [120]. The hybrid nanomaterial exhibited an improved OH functional group when compared to singular nanomaterial, while exhibiting impressive reusability for HM adsorption. On the other hand, Yarandpour et al. developed a unique mesoporous poly (acrylic acid) (PAA)/dextran-polyaniline (PANI) core-shell nanofiber via the spinning process [121]. The hybrid nanofibers exhibited a fibrous morphology with a flake-like structure, developing a highly porous network throughout the nanomaterial. Subsequently, the nanofibers were able to adsorb more than 95% of Pb (II) ions from aqueous solution. Another hybrid material consisting of zeolite and silica oxide (SiO_2_) was developed via wet chemical synthesis for the removal or HM in low concentrations [122]. The study suggests that both SiO_2_ and zeolite work in tandem, where the outer layer consisting of SiO_2_ rapidly adsorbs Zn (II) and Pb (II), and it then transports it via diffusion to its zeolite core, greatly enhancing the adsorption capacity. The employment of a non-metallic material that can produce a porous network was also the theme of the study done by Lui et al., where a biomimetic SiO_2_@chitosan composite was effectively able to adsorb As (V) and Hg (II) when compared to singular nanomaterial [123].

### 4.3. Synthesis and Modification of Nanomaterials

**Figure 6:**
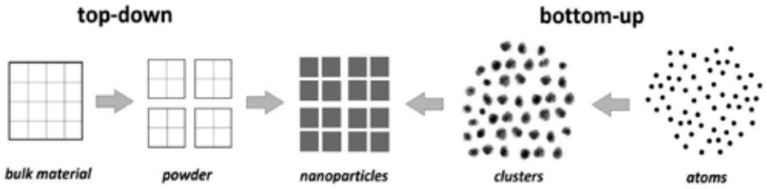
Nanomaterial synthesis route of nanomaterials following top-down, or bottom-up. Adapted from [107], with permission from CHEMIK, 2014.

The synthesis technique that was chosen for the formation of nanomaterials plays a significant role in the final characteristics exhibited. Generally, the synthesis route of nanomaterials follows two ways, which are top-down or bottom-up [110]. Figure 6 illustrates the building mechanism of the nanomaterial on both routes.

The synthesis technique controls the design and building mechanism of nanoparticles to produce materials of precise structure, shape, and size. Some of the synthesis technique includes wet chemical synthesis, the solution combustion technique, hydrothermal technique, sol-gel technique, and mechanical milling [110]. However, there are three prominent methods i.e., sol-gel, hydrothermal, and wet chemical synthesis. These methods are prominent due to the fact that they are easily produced and facile, do not require expensive equipment’s, and are highly reproducible. Sol-gel is one of the cheapest ways to produce nanomaterials with precise control on the stoichiometry of nanomaterial. The nanomaterials that were synthesized in this method generally had a small particle size and narrow size distribution due to their linear growth rate across the gel. As the name suggests, the precursors, commonly being metal alkoxides or metal chlorides, are hydrolyzed with water and alcohol and are then mixed and allowed to form a gel-like structure, before proceeding with calcination or sintering to remove the gel structure, leaving defined nanomaterials. Luu et al. synthesized a hybrid material that consisted of iron and TiO_2_ via the sol-gel technique. The formed nanoparticle exhibited a size of 19.5 nm and a surface area of 42.9 m^2^/g [124]. Another study for the formation of the same nanomaterial that was produced by Luu et al. exhibited a particle size of in the range of 19 to 7 nm and a surface area of between 84 and 182 m^2^/g. The results indicated that the particle size highly governed the surface area, where smaller nanoparticles possessed a larger surface area [125]. Aware et al. produced a hybrid material of Zn and TiO_2_ via the sol-gel method. The nanoparticle that was produced exhibited a particle size in the range of 12.6 to 18.1nm and a surface area value between 43.376 and 63.667 m_2_/g [126]. Another popular method in nanomaterial synthesis is a hydrothermal technique. This is a versatile and simple method in the synthesis of nanomaterial under the high pressure and temperature closed condition. Precursors are commonly stirred with a strong alkali solution (NaOH) and are stirred until the precursors have reacted with the alkali reagent. The materials are then transferred into a Teflon lined autoclave stainless steel block and reacted under a predetermined temperature and time. The produced sample will then be washed with water to remove the excess sodium hydroxide (NaOH), and then dried and ground to obtain a high yield of nanomaterials. This method is popular because of its high degree of controllability [127]. Nanomaterials of different physical properties, including size, structure, and surface area can be easily obtained by small tweaks in the synthesis parameters. TiO_2_, which has a globular shape, can be changed into TiO_2_ nanotubes via the hydrothermal method, increasing its surface area from 40 m^2^/g to 200 m^2^/g [128]. Hydrothermal can also be used to produce hybrid nanomaterials in a facile way. The versatility of the hydrothermal technique allows it to form metal and non-metallic hybrid nanomaterials. Zhang et al. produced a hybrid nanomaterial that consists of Ag and nanocellulose, which exhibited high bactericidal efficiency against both bacteria and fungus with an average nanoparticle size of 86 nm [129]. Hydrothermal can also provide an alternative for synthesis of novel material, such as graphene oxide (GO), which is commonly produced via the Hummer’s method [130]. In line with the simplicity of the synthesis method, wet chemical synthesis, or also known as the liquid phase synthesis technique, has also garnered the interest of researchers due to its simplicity while providing a large degree of control on the final physical characteristics of nanomaterials produced [131]. The process commonly involves the mixture of solutions of different ions at quantified volumes or molarity and provided a controlled amount of heat to initiate the formation of nanomaterials via precipitation. The excess solution is then washed away, and the precipitated nanomaterial is dried and ground/milled. This method offers control in nanomaterial stoichiometry, similar to the sol-gel method, and it can be conducted at a low temperature, reducing the energy consumption [131]. Table 3 shows some of the recent literature on the synthesis of hybrid nanomaterials using various techniques.

## 5. Recent Progress and Performance Evaluation

### 5.1. Adsorption of Heavy Metal

Research that was conducted by Mousavi et al. was successful in developing a novel bifunctional ordered mesoporous silica via wet chemical synthesis, creating a porous nanomaterial with a highly arranged network of compact silicone group (S-O) [138]. The developed nanomaterial was able to adsorb 98.6% of Cu (II) and 98.09% of Zn (II) ions. Sharma et al. successfully synthesized a hybrid ZnO and TiO_2_ monolith via the nanocasting technique. The hybrid material was able to adsorb Cd (II) via monolayer adsorption with an adsorption capacity of 786 mg/L, as compared to 643 mg/L for ZnO only monolith [83]. Castro et al. produced a biogenic iron compound via HM adsorption using biogenic iron sourced from mining wastewater that was able to absorb As (97.9 mg/L), Cr (20.1 mg/L), Zn (60.3 mg/L) and Cu (95.5 mg/L) in the optimum condition [84]. Activated carbons that were produced from nutshell removed almost 100% of Pb (II), 90–95% of Cu (II), and 80–90% of Zn (II), while biochar that was produced by pyrolysis of waste sludge was able to remove Cu (II), Cd (II), and Pb (II) by more than 90%. Based on the current research trends, researchers are exploring new and environmentally friendly carbon source to produce nano-sized carbon-based adsorbents that can improve HM adsorption due to the key features, such as the high surface area and porosity, ease for surface alkali functionalization, and being economically and environmentally viable. Deng et al. prepared a review on the utilisation of waste, such as palm shell, waste plastic, biomass, and so on to develop carbon based nanomaterials, including CNT and graphene [139]. Another emerging trend among research in the development of efficient adsorbents are the fabrication of various hybrid materials, which is expected to provide a synergistic effect in enhancing particle HM adsorption capacity. Kumar et al. developed hybrid carbon nanofibers and TiO_2_ polyacrylonitrile (PAN) membranes via electrospinning to adsorb HM ions, such as Pb (II), Cu (II), and Cd (II) [140]. The developed CNFs/TiO_2_–PAN hybrid membranes exhibited maximum adsorption of around 87%, 73%, 66%, for Pb (II), Cu (II), and Cd (II) metal ions, respectively. Another hybrid material comprised of Fe_3_O_4_ and *Raphiafarinifera*, a mangrove plant developed via the chemical co-precipitation method exhibited superior adsorption for Pb (II), Cu (II), Ni (II), Zn (II), and Cd (II) [141]. The hybrid material showed greater HM ion adsorption when compared to the singular particles, exhibiting the synergistic effect of the hybrid nanomaterial. Jiang et a. developed a hybrid graphitic carbon nitride nanosheet for the adsorption of both cationic and anionic HMs from wastewater [89]. The maximum adsorption capacities of Cd (II), Pb (II), and Cr (VI) on the g-C_3_N_4_ nanosheets are 123.205 mg/g, 136.571 mg/g, and 684.451 mg/g, respectively. The developed adsorbent can also be reused up to 10 times while achieving an adsorption capacity of 80% or more in 10 cycles.

Table 4 tabulates the synthesis technique and features that are imparted due to the formation of nano-sized adsorbents, which includes improved electrical conductivity, partial cation exchange, highly selective adsorption, high particle porosity, and surface chemical activation. An increase in surface area allows for more adsorption sites, which directly increases adsorption capacity. Porous structure also exhibits the same feature, in which the availability of binding sites for HM ions increases with the increasing porous structures. Nanoparticles are also functionalized to produce highly selective adsorbents, which are capable of adsorbing particular HM ions in a mixed metal water system. Chen et al. demonstrated that, by doping SiO_2_ with polythiophene (PTh), the composite recorded an impressive selectivity towards Zn (II) ions in multiple ion solutions (with *p*-value > 0.8 as compared to 0.3 of Pb (II)), which has also increased the adsorption capacity [142]. It was postulated that PTh has an affinity towards Zn (II) ions, which contributed to the overall selectivity. These features are important in improving the adoption of cationic HMs, such as increasing the strength of adsorption and increasing the adsorption capacity due to the porous structure of nanoparticles as adsorbents. Additionally, literature showed that there are prominent metal ions that are consistently used as model ions, including Cu (II), Pb (II), Co (II), Cr (II), and Zn (II) [143]. Other notable HM ions that are studied upon include Li (I), As (II), and Ni (II). In regards to adsorption capacity, the results vary, which is expected when presuming that all of the nanomaterials produced have varying features that highly influence the adsorption of specific HMs. The employment of hierarchically mesoporous carbon was able to adsorb Cu (II) up to 215.0 mg/g, while PTh/SiO_2_ nanoparticles were only able to adsorb 35.3 mg/g of Cu (II) [142].

A hybrid nanoadsorbent consisting of Fe and GO was synthesized to produce a magnetic graphene oxide (MGO) [37]. The MGO exhibited superior adsorption of Cd (II) and As (V) as compared to singular GO. The superiority of MGO was mainly attributed to its high dispersibility, thin nanosheets that were exhibited by GO, the synergistic effect that resulted from the electrostatic attraction offered by Fe, and various O-containing functional groups due to the surface functionality of GO. Marciniak et al. synthesized oxidized mesoporous carbon nanoparticle via the hart template method for the adsorption of Ni (II) and Cd (II) [144]. The nanoparticles were oxidized in different degrees to vary its oxidizing functional group. The results indicated that the nanoparticle with highest surface functional groups of acidic character was able to adsorb more HM ions. Similarly, research that was conducted by Li et al., where they prepared a sludge-based activated carbon impregnated with HNO_3_ for the removal of Pb (II), was highly governed by the surface functional group [145]. The results indicated that modified adsorbent rich with carboxyl group (R-COO^−^) was able to adsorb up to 98% of Pb (II), as compared to an unmodified particle which was only able to adsorb 83% of Pb (II). Huang et al. prepared a core-shell Fe_3_O_4_@polytetramethylene terephthalate (PTMT) composite magnetic microspheres for the adsorption of HM in highly saline water [38]. Surface amine functionalization greatly reduced particle agglomeration, which was complicated by nanomaterials with large surface area, while the incorporation of magnetite enhanced the recovery efficiency of the adsorbent while using a simple magnet. In addition, the shell structure also increased the particle surface area and porosity, increasing the adsorption sites, which improved the adsorption capacity of Hg (II) and Pb (II). Evidence of the impact that unique structure has on the adsorption of HM can also be seen in the study done by Ma et al., where a waste cotton fabric based double network hydrogel was developed for HM removal [146]. The experimental data suggested that the porous and sheet-like laminar structures that were exhibited by the nanostructured adsorbent were the reason behind the fast kinetics of Cu (II) and Cd (II) sorption equilibrium displayed. Similarly, Wang et al. developed a hybrid graphene oxide/silk fibroin hybrid aerogel [147]. The synthesized hybrid nanomaterial exhibited a porous network, which helped the HM ions to easily diffuse into the aerogels, while the silk fibroin exhibited great chelating feature, holding onto the HM ions for excellent adsorption capacity (Ag (II): 195.8 mg/g, Cu (II): 72.1 mg/g, Cu (II): 83.4 mg/g). Table 4 shows a compilation of recent literature on the development of various types of adsorbent for the removal of various HM ions from wastewaters.

### 5.2. Photocatalysis of Heavy Metal

**Figure 7:**
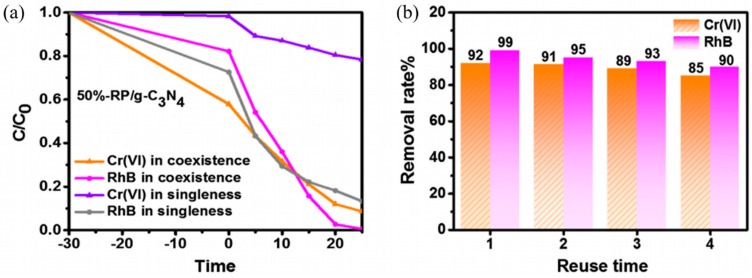
(**a**) Photoreduction of Cr (VI) in the presence and absence of Rhodamine B (RhB) and (**b**) removal rate of both Cr (VI) and RhB at different individual cycles. Adapted from [155], with permission from Elsevier, 2019.

Recently, an iron oxide (II) bismuth carbonate hybrid photocatalyst was developed and it showed excellent photocatalytic activity towards the reduction of carcinogenic and mutagenic Cr (VI) to nontoxic Cr (II) [151]. Another study that was done by Du et al. showed excellent photocatalytic Cr (VI) reduction to Cr (III) [152]. Kumar et al. employed a hybrid WO_3_/reduced graphene oxide (rGO) nanocomposites photocatalyst that exhibited a photocatalytic reduction of Cr (VI) following first-order kinetics and rate constants were found to be 0.0084 min^−1^ [153]. Even though traditional semiconducting photocatalyst exhibited good HM ion reduction, they are exclusively responsive towards dedicated UV light sources due to their large band gap. In order to overcome this, traditional photocatalyst were doped/modified with metallic or non-metallic materials to impart visible light sensitivity, producing nanocomposite photocatalyst. The presence of a froing atom on the surface photocatalyst also reduces the band gap, which reduces the energy that is required to excite an electron for photocatalyst initiation. It also increases the rate of photocatalysis due to the presence of a charge carrier [154]. This allows the photocatalyst to absorb photons from visible light sources to excite an electron and initiate the photocatalytic reaction. Figure 7 shows the photoreduction performance of Cr (VI) in a single system and in coexistence with an organic pollutant.

Lie et al. conducted a study on the simultaneous photoreduction of Cr (VI) and the photodegradation of Rhodamine B (RhB) to in an independent and dependent system. The results that are depicted in Figure 7a,b indicates that the photoreduction of Cr (VI) in the presence of RhB is significantly better when compared to photoreduction in a solution only containing Cr (VI) ions [155]. The research work for the simultaneous degradation of phenol and photoreduction of Cr (VI) that was conducted by Yu et al. explained that the presence of phenols improves the photoreduction of Cr (VI) as the electron-hole is consumed by phenol. This suppresses the recombination of electron and holes, which in turn accelerates the photoreduction of Cr (VI) [48]. The same reasoning has been used in other research, where the photoreduction of Cr (VI) was accompanied by the degradation of various types of organic pollutants to sustain the reaction, such as 2-Mercaptobenzothiazole (MBT) [156], sulfamethoxazole [157], RhB [98,136], and chlorinated phenols [158]. The photoreduction of both Pb (II) and Cu (II) was attempted together with the photodegradation of RhB while using a modified Chitosan-Gelatin @ zirconium (IV) selenophosphate nanocomposite ion exchanger. The hybrid photocatalyst was able to retain more than 90% of the HMs, while degrading more than 80% of RhB within 120 min [159]. Another example was exhibited in a research work that was conducted by Du et al. Their investigation showed that the addition of different organic compounds, like citric acid, oxalic acid, and diclofenac sodium mimics the function of a hole scavenger, which increases the photocatalytic Cr (VI) reduction activity. The ability of hole scavengers to consume the photoinduced holes that are produced by the photocatalyst upon light irradiation is accepted as a plausible reason. This allows for more electrons to escape from pair recombination and become available for the reduction of Cr (VI) ions [152]. Photocatalysis have also shown the potential to photoreduce Cr (VI) into Cr (III) while exhibiting potential to kill harmful bacteria, such as *E. coli* (89%) and *S. aureus* (81%) within 10 min of irradiation on photosensitized TiO_2_ nanofibers [160]. In recent years, the development of binary (bi-material) and ternary (tri-material) photocatalyst has intensified in search of combinations to produce photocatalyst that is responsive towards visible light, low recombination rates, and increased surface reactive sites.

An aggregation free CeO_2_/SnO_2_/rGO was developed via the hydrothermal process for the simultaneous degradation of MB and photoreduction of Pb (II) and Cd (II). The photocatalyst exhibited close to 100% degradation of methylene blue (MB) and a HM ion reduction of close to 80%. The rGo acts as a charge suppressor and, due to its excellent electrical conductivity, it can trap the electron that is excited from the conduction band (CB) of SnO_2_ and CeO_2_, allowing for longer photocatalyst activation. The exploitation of rGO, as an excellent charge separator and trapping site to sustain photocatalytic formation of ROS, was also explored by Bai et al., where a Red Phosporus (RP)-MoS_2_/rGO ternary photocatalyst was developed via a facile two-step hydrothermal [161]. RP and MoS_2_ exhibited excellent photocatalytic activity and increased the number of excited electrons/holes under visible light irradiation, while rGO was responsible for enhancing charge separation to sustain and provide stability in electron recombination. These factors synergistically assisted RP-MoS_2_/rGO to perform impeccably, reducing Cr (VI) by up to 95%. Another nanomaterial that exhibits charge trapping capability is carbon nitride (C_3_N_4_), which is a relatively new nanomaterial. Liu et al. developed a binary RP/g-C_3_N_4_ photocatalyst for visible light photoreduction of Cr (VI). The catalyst exhibited good stability for removing 85% Cr (VI) and 90% RhB, even after four times of recycling, owing to the effective separation and rapid transfer of e^−^/h^+^ pairs between RP and g-C_3_N_4_. With the low band gap exhibited (1.35 eV), ●O^2–^ can be generated in this system for the lower CB position of RP, where the excited electron can jump into the CB of RP, sustaining photocatalytic activity for a longer period. The synergistic redox reaction allows for Cr (VI) to reduce into Cr (III) [155]. Ye et al. managed to synthesize a ternary Ag/Bi_4_O_7_/g-C_3_N_4_ nanosheets Z-scheme heterojunction photocatalyst for the photoreduction of Cr (VI) while using a combination of thermal polymerization, hydrothermal, and calcination [162]. The structure exhibited the deposition of Ag and Bi_4_O_7_ on sheets of g-C_3_N_4_, similarly to the work that was conducted by Liu et al. The relatively low band gap of both Bi_4_O_7_ (1.89 eV) and g-C_3_N_4_ (2.97 eV) enabled the ternary photocatalyst to exhibit excellent visible light responsive capabilities. Additionally, the presence of g-C_3_N_4_ sheets offered excellent charge separation capabilities, thus enhancing the photoreduction of Cr (VI) [163]. Jing et al. developed a three-dimensional PANI/MgIn_2_S_4_ nanoflower photocatalyst via electrostatic adsorption between PANI and MgIn_2_S_4_. PANI is known to be a good polymeric electric conductor due to the presence of heteroconjugated π bond, making it a suitable candidate as a dopant for photocatalysts [164]. The ternary photocatalyst was able to reduce Cr (VI) by up to 95% within 15 min of light irradiation. The photogenerated electrons in the valence band of MgIn_2_S_4_ and the highest occupied molecular orbital (HOMO) of PANI could be excited to the corresponding conduction band and the lowest unoccupied molecular orbital (LUMO) when it assimilates photons with energy larger than the band gap, while the holes were generated in the valence band of MgIn_2_S_4_ and the HOMO of PANI. This interaction accelerated the photoreduction of Cr (VI) and the overall photoactivity [165]. Table 5 shows the development of nanocomposite photocatalyst for the reduction of various types of HM.

### 5.3. Membrane Composite for Removal of Heavy Metal

Nanomaterials are commonly added into polymeric membranes during preparation, where it is dispersed into the dope solution before the membranes are formed via the dry-wet phase inversion technique. The dispersion of nanomaterials across the membrane matrix or deposition on the membrane surface, as in the case of thin-film nanocomposite (TFN) membrane, has significantly altered the permeation and rejection performance of the membranes. Lakhotia et al. developed a TFN membrane with FeO nanoparticle dispersed on the membrane selective layer. As the concentration of FeO nanoparticles increased on the membrane surface, the hydrophilicity and surface charge of the nanocomposite membranes were effectively enhanced. The membrane flux was enhanced from 27.46 to 36.85 L/m^2^ h and high rejection of Mg (II) and Na (I) rejection (>90%), whilst membrane without the FeO nanoparticle rejected salt in the range of 65%. An AlTi_2_O_6_ incorporated polysulfone (PSF) composite membrane exhibited improved hydrophilicity with the water contact angle being reduced from 73° to 51° [173]. The presence of AlTi_2_O_6_ hindered the flow of non-solvent during membrane casting, which created more membrane pores on the surface. Polypyrrole (PPy)@Al_2_O_3_ was added into a polyethersulfone (PES) membrane matrix for the removal of Cu (II). The addition of PPy@Al_2_O_3_ improved the membrane water transport capacity, reduced membrane surface roughness, and eventually mitigated membrane fouling [174]. The nanocomposite membrane also exhibited improved Cu (II) rejection (25% to 81%). Ghaemi et al. developed a polyaniline modified GO nanoparticle that was incorporated into a PES membrane for the remediation of water laden with Pb (II), which also showed good metal ion removal because the incorporating of PANI modified GO increased the viscosity of the membrane dope solution, which in turn reduced the mean pore size of nanocomposite membranes [175]. The filtration of HM-laden water produced clear water containing trace levels of HM as permeate, in which the HMs were concentrated in the retentate.

A study done by Nasir et al. showed that the incorporation of hydrous iron manganese nanoparticle into a PSF membrane was able to adsorb As (II) up to 4.1. mg/g [176]. A zirconia polyvinylidene fluoride (PVDF) composite membrane that was developed by Zheng et al. was able to adsorb As (V) at 25.5 mg/g, whilst maintaining membrane flux of 177.6 L/m^2^ h [69]. Another work that was done by He et al. incorporated zirconia into a PSF hollow fiber membrane for the removal of As (V) [177]. This work exhibited a much higher adsorption capacity of 131.8 mg/g at zirconia/polymer ratio of 1.5. In a separate study, zirconia was modified while using phosphate solution to produce a PVDF membrane with surface coated nanomaterial that performed excellently for the removal of Pb (II) ions [178]. The adsorption capacity that was recorded by the optimized membrane was 121.2 mg/g at the pH of 5.5. The modification also allowed for selectively improving the membrane adsorption to favor Pb (II) over Zn (II) ions. Delavar developed HMO nanoparticle incorporated membrane for the removal of Cd (II) and Cu (II), where it was revealed that the loading of nanomaterial into the membrane matrix largely influences the adsorption performance. The polymeric structure of the membrane only serves as a pathway for untreated water to pass and to immobilize the adsorbents. Membrane that was loaded with 3 wt% adsorbed 31.3 mg/g of Cd (II) and 29.7 mg/g of Cu (II), whilst 10% loaded membrane adsorbed 33.18 mg/g and 30.6 mg/g of Cd (II) and Cu (II), respectively, asserting this notion [179]. In addition to this, another work on the employment of HMO as a nanoadsorbent, immobilized in a PES membrane, was able to adsorb Pb (II) at an uptake capacity of 204 mg/g, highlighting the potential of HMO as an excellent candidate for HM adsorption [180]. Table 6 shows the details of membranes incorporated with nanomaterials that were developed for the rejection of HMs.

## 6. Conclusions and Future Perspective

The advances of nanomaterial in terms of development of materials with novel structure, characteristics, and hybrid nanomaterials have further revealed unique and amplified properties. The ability to develop nanomaterials atom by atom with highly controllable methods is behind the rise of novel nanomaterials. The review highlights the roles of nanomaterials in heightening the efficiency of the above-mentioned technologies for HM removal. However, there is plenty of room for future development and research to discover new hybrid nanomaterials for the removal of HM from wastewaters. Removing HM ions from water sources is a critical task. Removing and destroying HM is difficult due to its stability in ionic form. However, recent advances in using various types of nanomaterials that were developed with HM removal indicate the potential that researchers see in nanomaterials. Of all the proven methods to remove HM, three prominent ways i.e., adsorption, photocatalysis, and membrane separation have been comprehensively reviewed. Undeniably, nanomaterials have enabled significant breakthrough that was made in these strategies. For the adsorption of HM, researchers have developed plenty of nanomaterials, singular or hybrid, which work as nanoadsorbent to trap HM from water bodies, as displayed in Table 4. Even though all of the nanomaterials excel as absorbents, more emphasis needs to be placed in the development of functional HM adsorbents using waste products via the facile synthesis method. The reason behind this notion is that greater research emphasis is required to produce nanomaterials that can be easily scaled up for real-life usage. For instance, producing a very large amount of absorbents for effective treatment of large water bodies, such as lakes and rivers. Current trends in developing adsorbents from waste, such as from biochar or sludge waste, can be studied in depth in terms of upscaling. It needs to be highlighted that, whilst it is great for researchers to pursue unique and novel nanomaterial with new features that can aid in HM removal, it is important to consider the effectiveness of the approaches for the large-scale removal of HM. Photocatalysis has shown great promise in reducing HM ions while destroying organic pollutants, which exhibits the versatility of semiconducting nanomaterial as the total remediation of wastewater. Great strides have been taken in sensitizing traditional semiconductors to respond towards visible light source by doping. However, doping needs to be focused on utilizing non-noble metals and non-metal dopants, which are much more abundant in nature when compared to rare and noble metals. In addition, future research to be conducted can place better emphasis on the doping method to improve the precision of dopant incorporated into semiconductor nanomaterials. On the other hand, the feasibility of employing plasmonic metal nanomaterial for the reduction of HM ions should also be explored. Plasmonic metal nanoparticles are known as light harvesting materials that exhibit the ability to harvest visible light photons through the excitation of localized surface plasmon resonance (LSPR). Currently, the studies on this nanomaterial to reduce HM ions in waterways are very scarce. The unique characteristic of the plasmonic metal nanoparticle is expected to spur interests in this field [188,189]. The incorporation of functional nanomaterials has enhanced polymeric membrane separation and permeation efficiency, where membrane separation or adsorption are two viable ways of removing HM ions from water sources. However, the agglomeration of nanomaterial in the membrane matrix is a common problem, which limits the loading of nanomaterial and reduces the efficiency of nanomaterials. To counter this, steps to functionalise the nanomaterial surface to inhibit agglomeration (employing silane agents, improved surface charge) is welcome progress in further maximizing the synergistic of nanomaterial and membrane technology.

## Figures and Tables

**Table 1:** Maximum level of heavy metal (HM) content in water samples. Table was reproduced from [6], with permission from EDP Sciences, 2017.

Agency	Permissible Level of HM (mg/g)								
	Cd (II)	Cr (III)	Co (II)	Cu (II)	Pb (II)	Fe (II)	Mn (II)	Hg (II)	Ni (II)
National Agency for Food and Drug Administration and Control (NAFDAC)	0.0	NM	NM	NM	0.0	NM	NM	0.0	NM
United States Environmental Protection Agency (USEPA)	0.005	0.1	0.1	1.3	0.015	0.3	0.05	0.002	0.1
World Health Organization (WHO)	0.003	NM	NM	0.01	0.01	0.3	0.4	0.001	0.07
Department of Environment (DOE), Malaysia	0.005	0.05	NM	1.0	0.1	1.0	0.2	NM	NM

Remark: NM refers to ‘not mentioned’.

**Table 2:** Types of nanomaterials and the important features they exhibit as compared to bulk material.

Material	Classification	Unique Feature	Synthesis Technique	Application	Reference
Streptavidin (SA)-horseradish peroxidase (HRP)	Nanoflowers	Improved biocompatibility and attachment of the protein	Wet chemical synthesis	Biomarker detection	[95]
TiO_2_ and diatomite	Nanoparticle	High surface area, improved absorbability	Wet chemical precipitation	Photocatalyst	[96]
Fe_3_C	Nanoparticle	Good heating ability in magnetic fields	Hydrothermal and sonication	Magnetic hyperthermia	[97]
BiOBr/Ti_3_C_2_	Nanoparticle	Surface functionalisation	Self-assembly	HM photoreduction	[98]
ZnO	Nanorods	Improved electrode performance	Hydrothermal and sputtering	Energy nanogenerators	[99]
Au	Nanorods	Huge electric field enhancements	Direct growth and fabrication	Plasmonic spectroscopies	[100]
Ag	Nanorods	Increased dispersion and stability	Wet chemical synthesis	Transparent heaters	[101]
TiO_2_	Nanotubes	High hydrophilicity, surface area	Hydrothermal	Membrane filler	[102]
SiO_2_-Ge	Nanotubes	Excellent thermal transport, large surface-to-volume ratio.	-	Phonon transport	[103]
Carbon	Nanotubes	High surface area and adsorption capacity	-	Adsorption of diazinon	[104]
Cu_3_(PO_4_)_2_.	Nanoflowers	Spherical, porous and hierarchical structure	Wet chemical synthesis	Photodegradation of phenol	[105]
WS_2_	Nanoflowers	Increased reaction sites	Hydrothermal and reduction	Hydrogen generation	[106]

**Table 3:** Synthesis of hybrid nanomaterials using different techniques.

Product	Materials	Method	Parameters	Nanomaterial Characteristics	Reference
Mn-*g*-C_3_N_4_	Mn and *g*-C_3_N_4_	Combustion	Heated at a rate of 520 °C with a rate of 4 °C/min (2 h)	Improved ROS creation, lower band gap (1.25 eV)	[132]
Ag-GO	Ag and reduced GO	Modified Tour’s method	Oxidized under 15 °C, heated to 50 °C, washed and freeze-dried	Visible light absorption improved oxidant generation capacity	[133]
CNT@MoS^2^/SnS_2_ nanotubes	CNT, MoS_2,_ and SnS_2_	Hydrothermal	Autoclave for 180 °C for 20 h washed with water	Faster reduction of Cr (VI), the narrow bandgap	[134]
SnO_2_-SrO	SnO_2_ and SrO	Sol-gel	Gel formed, digested and dried in an oven at 100 °C, washed with ammoniated water	Lower band gap (2.23 eV, impart gas sensing	[135]
BiOBr/Ti_3_C_2_	BiOBr Ti_3_C_2_	Self-assembly method	BiOBr and Ti_3_C_2_ co-precipitated under magnetic stirring	Visible light photodegradation, surface reactivity	[98]
Yttrium/H-titanate	Yttrium and TiO_2_	Hydrothermal	Ti(SO_4_)_2_ and hydrazine hydrate (N_2_H_4_·H_2_O) reacted in an autoclave for 130 °C	Reduction in the efficiency of charge separation	[136]
TiO_2_-MgO	(titanium isopropoxide and magnesium methoxide	Sol-gel	Sol-gel formed, dried at 100 °C and calcined at 900 °C	Visible light sensitivity, uniform hybrid material	[137]

**Table 4:** Recent literature on the development of various types of adsorbent for the removal of HM ions from wastewaters.

Nanomaterial	Synthesis Technique	Features	Metal Species	Adsorption Capacity	Optimum pH	Reference
Mesoporous carbon	Hard template technique	High uniformity of porous structure, surface functionalized	Ni (II)	140.9 mg/g	5	[144]
			Co (II)	129.9 mg/g		
Hierarchically porous carbon	Pyrolysis and chemical activation	KOH activated	Cd (II)	180.0 mg/g	6	[40]
			Pb (II)	220.0 mg/g		
			Cu (II)	215.0 mg/g		
			Zn (II)	95.0 mg/g		
			Cr(III)	140.0 mg/g		
Geopolymers	Alkali activation of aluminosilicate	Alkali activated, inorganic polymers	Ni (II)	85.3 mg/g	10	[148]
			Pb (II)	111.0 mg/g		
			Cd (II)	130.5 mg/g		
Polyaniline/TiO_2_	Chemical oxidative polymerisation	Self-doping, highly selective adsorption	Zn (II)	51.6 mg/g	5	[45]
			Pb (II)	96.2 mg/g		
			Cu (II)	18.2 mg/g		
Ga-doped ZnO	Sol-gel	Improved electrical conductivity	Cr (II)	52.2 mg/g	3–5	[149]
			Cd (II)	28.3 mg/g		
Polythiophene/SiO_2_	Sol-gel	Stable and highly selective	Pb (II)	70.9 mg/g	5	[142]
			Cu (II)	35.3 mg/g		
			Zn (II)	34.6 mg/g		
Zeolite	Amino acid as mesoporogens	Partial cation exchange	Cu (II)	171 mg/g	11	[46]
			Ni (II)	99.1 mg/g		
			Pb (II)	514.0 mg/g		
Wild herb nanoparticle	Ball milling	Environmentally friendly	Cd (II)	52.9 mg/g	12	[39]
			Co (II)	40.8 mg/g		
			Li (II)	181.8 mg/g		
Fe_3_O_4_	Carbon microsphere	High BET surface area, hierarchical as well as mesoporous structures	Pb (II)	95.2%	6	[150]
			Cd (II)	96.2%		
			Cr (III)	98.2%		

**Table 5:** Development of nanocomposite photocatalyst for the reduction of various types of HM.

Photocatalyst	Dopant	Method	Metal Species	Removal Performance	Reference
Ag/Bi_4_O_7_/	g-C_3_N_4_	Thermal polymerization, hydrothermal and calcination	Cr (VI)	90%	[162]
WO_3_	Reduced graphene oxide (rGO)	In-situ hydrothermal	Cr (VI)	90%	[153]
Fe_2_O_3_	Bismuth carbonate (BOC)	Two-step chemical modification	Cr (VI)	>90%	[151]
TiO_2_	Graphene	Hydrothermal	Pb (II)	60%	[166]
V_2_O_5_ nanorod	g-C_3_N_4_ nanosheets	Facile impregnation	Cr (VI)	71%	[167]
Zirconium	Selenophosphate	Two-step ion exchanger	Pb (II)	100%	[159]
			Mg (II)	95%	
Red phosphorus	g-C_3_N_4_ nanosheets	Thermal polymerization and hydrothermal	Cr (VI)	92%	[155]
TiO_2_	-	-	Cd (II)	98%	[168]
			Pb (II)	99%	
Metal organic framework 100	g-C3N4 nanosheets	Calcination and hydrothermal	Cr (VI)	98%	[152]
Zn	Coordination polymers (H_2_L and by)	Hydrothermal	Cr (VI)	100%	[169]
CdS	CuInS	Hydrothermal	Cr (VI)	100%	[170]
Titanate nanosheets	Yttrium	Hydrothermal	Cr (VI)	>75%	[136]
TiO_2_	Graphene	Hydrothermal	Zn (II)	100%	[171]
TiO_2_	Graphene	Hydrothermal	Pb (II)	>70%	[166]
CeO_2_/SnO_2_/	rGO	Hydrothermal	Pb (II) Cd (II)	80% 80%	[172]

**Table 6:** Membranes incorporated with nanomaterials for HM ion removal.

Polymer	Nanomaterial	Removal Method	Metal Species	Control Membrane Performance	Composite Membrane Performance	Reference
Polyethylene oxide (PEO)	Halloysite nanotubes and	Adsorption	Cr (VI)	80 mg/g	85 mg/g	[181]
			Cd (II)	105 mg/g	115 mg/g	
			Cu (II)	120 mg/g	135 mg/g	
			Pb (II)	145 mg/g	155 mg/g	
Ceramic	Rice husk ash	Adsorption and Filtration	Ni (II)	-	99.99%	[182]
			Zn (II)		99.97%	
			Pb (II)		99/99%	
Polyvinyl chloride (PVC)	Carboxylated CNT	Filtration	Zn (II)	48%	93%	[183]
PVDF	Superhydrophilic alumina	Filtration	Pb (II)	84%	92.5%	[184]
Alumina Substrate	Zeolite imidazolate framework-30	Filtration	Cu (II)	-	99.87%	[185]
Polyethylenimine (PEI)	GO	Filtration	Zn (II)	0%	96.6%	[186]
Poly(ethyl methacrylate) PEMA	Rhodanine	Adsorption	Ag (II)	-	65%	[65]
			Pb (II)	-	58%	
PSF	Nickel/Iron oxide	Filtration	Pb (II)	-	95%	[65]
			Cu (II)	-	95%	
PAN	HMO	Filtration	Cu (II)	37%	70%	[187]
PES	Polydopamine	Adsorption	Pb (II)	>1 mg/g	20.3 mg/g	[56]
			Cu (II)	>1 mg/g	10.4 mg/g	
			Cd (II)	>1 mg/g	17 mg/g	
PSF	Al-Ti_2_O_6_	Filtration	As (II)	-	96%	[173]
			Cd (II)	-	98%	
			Pb (II)	-	99%	
PSF	Quaternized polyelectrolyte complex	Filtration	Mg (II)	86.4%	95.7%	[57]
			Zn (II)	87.1%	98.3%	
			Cu (II)	80.3%	97.9%	
